# Mutual plant-fungi symbiosis compromised by fungicide use

**DOI:** 10.1038/s42003-022-04029-w

**Published:** 2022-10-07

**Authors:** Gavin Duley, Emanuele Boselli

**Affiliations:** Faculty of Science and Technology, Free University of Bozen-Bolzano, Piazza Università 1, 39100 Bolzano, Italy

## Abstract

Soil microbiota, including arbuscular mycorrhizal fungi (AMF), are critical for plant nutrition in non-agricultural ecosystems. A new study by Edlinger et al. shows that agricultural soils are negatively impacted by fungicide use and generally have lower AMF diversity and abundance.

**Figure:**
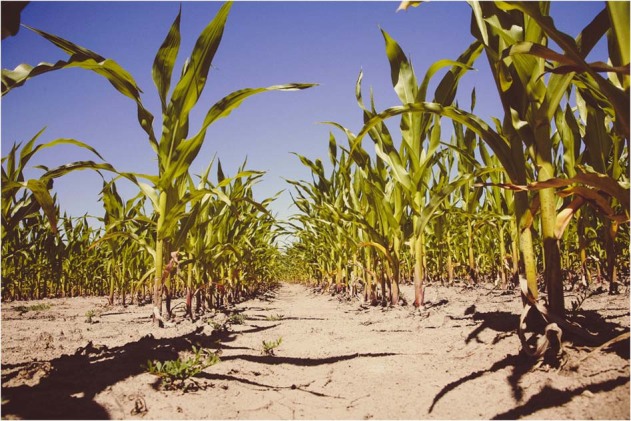
Markus Spiske

Agricultural productivity is an increasingly important topic as the global population increases, and the security of food supplies becomes more precarious. However, soil health is negatively impacted both by intensive farming and by climate change, in part due to altered microbial and fungal biodiversity^1^. Arbuscular mycorrhizal fungi (AMF) are found in the soils of most terrestrial ecosystems, where they facilitate nutrient uptake by (and exchange between) plants. Plants provide AMF in the soil with sugars and lipids, and receive nutrients such as nitrogen and phosphorus from AMF in return. In nutrient-poor soils, AMF may provide the majority of the nutritional requirements of host plants^1^.

A recent study led by Marcel G. A. van der Heijden at Agroscope and the University of Zürich (Edlinger et al.^1^) found that AMF diversity was lower in cereal-producing farmland soils than in grassland soils, because AMF are negatively impacted by agricultural practices such as plowing or the use of fungicides and inorganic fertilizers^1^. Other studies observed similar reductions in microbial and fungal diversity in the soils of other agricultural systems, such as vineyards^2,3^, orchards^3^, and arable crops^4^. Most modern crop plants have not been bred with this symbiotic relationship with AMF in mind, and thus are only inefficiently colonized by AMF^1^.

The study by Edlinger et al*.*^1^ examined the growth of plants in grassland, cultivated (arable farmland), and sterile control soil, using *Plantago lanceolata* L. (Plantaginaceae) as a model species. A genomic analysis was used to characterize the microbial diversity present in the soil, and nutrient (phosphorous) transfers were analyzed in a compartment system using the radio-isotope, ^33^P. The authors found considerable differences between the farmland and grassland soils, with lower hyphal ^33^P transfer in the farmland soil. In contrast, AMF communities in the grassland soil were able to compensate for low phosphorus availability. While other key factors like soil pH, soil organic content, and climate/aridity can inhibit AMF growth, the application of fungicide had the most critical impact on hyphal ^33^P transfer. In a separate experiment, the authors also modeled how AMF communities adapted to soil conditions typical of phosphorus-based fertilizers, observing that AMF communities were less active in the presence of higher levels of phosphorus. Altogether, the authors suggested that soil AMF could be beneficial to agricultural systems by providing crops with nutrients (especially phosphorus) without the negative environmental impact associated with high levels of phosphorus-based fertilizers^1^.

Intensive agriculture has improved productivity in many agricultural systems and allowed countries such as India to achieve agricultural self-sufficiency. However, little attention has been paid to soil health, including soil microorganism abundance and biodiversity^5^. Some years ago, viticultural consultants Claude and Lydia Bourguignon anecdotally warned Burgundy winegrowers that their soils had no more biological activity than the sandy soils of the Sahara^6^. While this suggestion may be somewhat overstated, the warning was prescient and has led to a reimagination of soil management practices in viticulture. Work like that of Edlinger et al*.*^1^ and Quiquerez et al*.*^2^ show the critical and broad importance of healthy soil and of the abundance and diversity of microorganisms in the soil for future agricultural systems. Future research could focus on agricultural methods that can improve the beneficial symbiosis between microorganisms such as AMF in the soil and plants while maintaining yields, with the possibility of partially replacing phosphorus-based fertilizers with AMF-mediated phosphorus uptake. However, it is unclear to what extent cultivated plants can benefit from these symbiotic relationships. Edlinger et al. suggest that the breeding and use of crops that can form these associations with AMF may be a beneficial direction for farming in the future^1^.

## References

[1] Edlinger A (2022). Agricultural management and pesticide use reduce the functioning of beneficial plant symbionts. Nat. Ecol. Evol..

[2] Quiquerez A (2022). Legacy of land-cover changes on soil microbiology in Burgundy vineyards (Pernand-Vergelesses, France). OENO One.

[3] Viti C (2008). Characterizing cultivable soil microbial communities from copper fungicide-amended olive orchard and vineyard soils. World J. Microbiol. Biotechnol..

[4] Helgason T (1998). Ploughing up the wood-wide web?. Nature.

[5] Singh RB (2000). Environmental consequences of agricultural development: a case study from the Green Revolution state of Haryana, India. Agric. Ecosyst. Environ..

[6] Capalbo, C. Claude and Lydia Bourguignon: famous chateau secrets. *Decanter*. https://www.decanter.com/features/claude-and-lydia-bourguignon-famous-chateau-secrets-247201/ (2008).

